# Percolation-Regime Modulation of Charge Transport and Humidity-Driven Conductivity in 3 wt.% Graphene Oxide/Carboxymethyl Cellulose Membranes

**DOI:** 10.3390/nano16120750

**Published:** 2026-06-15

**Authors:** Tilek Kuanyshbekov, Adilet Dautov, San Orazova, Ahmed Abdala, Zhandos Tolepov, Amantur Umarov, Roza Aubakirova, Batima Tantibaeva, Zhazira Mukazhanova, Yerkezhan Abikak, Bakhyt Shaikhova

**Affiliations:** 1 Department of Chemistry, Sarsen Amanzholov East Kazakhstan Univeristy, 30th Guards Division Str., 34/1, Ust-Kamenogorsk 070000, Kazakhstan; a.dautov.y@gmail.com (A.D.); sansovetbekovna@gmail.com (S.O.);; 2 Kaz Graphene, 63 Zapadnyi Str., Ust-Kamenogorsk 070011, Kazakhstan; 3 College of Science and Engineering, Hamad bin Khalifa University, Education City-Gate 8, Ar-Rayyan P.O. Box 5825, Qatar; communications@hbku.edu.qa; 4 Faculty of Physics and Technology, Al-Farabi Kazakh National University, 71 Al-Farabi Ave., Almaty 050040, Kazakhstan; 5 Prototyping and Incubation Center, Rudny Industrial University, 50th Anniversary of October Street, 38, Rudny 111500, Kazakhstan; 6 Institute of Metallurgy and Ore Beneficiation, St. Shevchenko, 29/133, Almaty 050010, Kazakhstan

**Keywords:** humidity sensor, biopolymer nanocomposites, proton conduction, interfacial interactions, electrical resistivity

## Abstract

This study investigates graphene oxide/carboxymethyl cellulose composite membranes containing 3 wt.% graphene oxide. The influence of the carboxymethyl cellulose content on the structural organization, mechanical properties, electrical resistivity, and humidity-dependent conductivity was systematically analyzed using Fourier transform infrared spectroscopy, scanning electron microscopy, X-ray diffraction, tensile testing, and electrical measurements. Fourier transform infrared spectroscopy indicated intermolecular interactions between graphene oxide and carboxymethyl cellulose functional groups. X-ray diffraction analysis showed gradual inter-layer expansion from 0.71 to 0.87 nm together with crystallite size reduction after polymer incorporation. Scanning electron microscopy observations demonstrated the increasing structural uniformity and polymer encapsulation of graphene oxide sheets with the increasing carboxymethyl cellulose content. Mechanical testing revealed improvement in the tensile strength from 6.6 to 17.8 MPa with the increasing carboxymethyl cellulose concentration. Simultaneously, the dry-state electrical resistivity increased from 5.8 × 10^6^ to 2.32 × 10^7^ Ω·m due to increasing dielectric separation between graphene oxide domains. Humidity-sensing experiments demonstrated reversible resistance changes in the 20–90% relative humidity range, associated with proton-assisted conduction through adsorbed water layers. The obtained results demonstrate that polymer incorporation strongly influences both the structural organization and electrophysical behavior of graphene oxide/carboxymethyl cellulose composite membranes.

## 1. Introduction

Graphene oxide (GO)-based polymer nanocomposites have attracted considerable attention as multifunctional materials for flexible electronics, membrane technologies, sensing systems, and sustainable structural applications. Unlike pristine graphene, GO contains a high density of oxygen-containing functional groups (hydroxyl, epoxy, and carboxyl), enabling strong interfacial interactions with polymer matrices and facilitating solution-based processing [1,2]. These functional groups allow GO to act not only as a reinforcing nanofiller but also as a structural regulator capable of modulating inter-layer spacing, stress transfer efficiency, and charge transport behavior.

In polymer nanocomposites, performance enhancement critically depends on filler dispersion, interfacial adhesion, and the formation of conductive or stress transfer networks. Mechanical reinforcement arises from improved stress redistribution within the composite structure [3,4,5,6,7,8,9,10,11] across the filler–matrix interface, while electrical transport emerges when partially continuous pathways form within the composite. For GO/polymer systems containing 2–5 wt.% filler, tensile strength improvements of 30–80% have been widely reported, depending on the dispersion quality and matrix compatibility [2,12].

The electrical properties in such systems typically range from highly insulating behavior (~10^−12^ S·cm^−1^) for poorly connected GO domains to semi-conductive regimes (~10^−6^–10^−3^ S·cm^−1^) when partial percolation is achieved, with percolation thresholds often reported between 1 and 4 wt.% GO [2,13,14]. In terms of resistivity, this corresponds approximately to 10^3^–10^8^ Ω·m depending on the oxidation degree and structural organization—values consistent with the ranges measured in the present study.

Recent studies demonstrate that GO/polymer composites near the 2–4 wt.% loading regime may exhibit changes in charge transport and mechanical behavior depending on the filler dispersion, polymer matrix, and interfacial organization [15,16,17,18,19,20,21]. Electrical conductivity values reported for GO/biopolymer systems depend strongly on the oxidation degree, dispersion quality, and structural organization of GO domains within the polymer matrix [15,16,17]. Tensile strength improvements have been reported in well-dispersed graphene/polymer systems, where the mechanical behavior is influenced by the filler dispersion, interfacial interactions, and polymer matrix compatibility [3,4,5,6,7,8,9,10,11], indicating that this concentration window represents a structurally sensitive region where interfacial interactions and filler network formation begin to overlap.

Humidity-sensitive GO composites further demonstrate resistance variations of 10^2^–10^4^ under 20–90% RH cycles [20,21,22,23]. However, many systems suffer from limited reversibility, structural instability, or mechanical degradation at higher polymer loadings. At elevated humidity, water adsorption in GO-based membranes can facilitate proton-assisted conduction through hydrogen-bonded water networks and confined inter-layer pathways [20,24,25,26]. Nevertheless, most existing studies evaluate structural, mechanical, and humidity-driven electrical properties independently.

Despite extensive studies on GO/polymer composites, the relationship between their structural evolution, interfacial interactions, mechanical reinforcement, and electrical behavior remains insufficiently understood, particularly for systems approaching the lower percolation region.

In many reported studies, structural characterization and functional properties are discussed separately without establishing direct correlations between crystallographic changes, morphology, mechanical performance, and humidity-dependent electrical response. Therefore, further investigation is required to clarify how polymer incorporation modifies the layered GO structure and influences the combined mechanical and electrophysical properties of GO/CMC composite membranes.

Most reported studies treat structural characterization and functional performance separately, without establishing a quantitative structure–property–function relationship.

Various structural platforms have been explored for the fabrication of polymer-based composite materials, including layered GO/polymer systems and porous anodic aluminum oxide (AAO) templates. In AAO-based composites, pore geometry, interfacial organization, and confinement effects can significantly influence the structural and functional properties of the resulting materials [21]. These studies further demonstrate the importance of controlling the microstructural organization for achieving the desired material performance.

Biopolymer matrices, particularly carboxymethyl cellulose (CMC), offer an environmentally sustainable platform for GO incorporation. CMC contains abundant −OH and −COO^−^ groups capable of forming hydrogen-bonded networks with GO sheets [12,27]. Such interactions improve dispersion and mechanical stability; however, the transition from hydrogen-bond-dominated reinforcement to filler-network-modulated electrical transport remains insufficiently clarified, particularly near the lower percolation regime. In addition, graphene oxide-based composite materials have demonstrated strong potential for environmental applications, including water purification systems. For example, GO-based filtration materials have been developed and patented for water treatment applications [13].

In our previous study [27], GO/CMC composite membranes containing 1 wt.% GO were synthesized from locally sourced graphite. That work demonstrated structural compatibility and moderate mechanical enhancement; however, the GO content was below the commonly reported percolation region. Consequently, inter-layer coupling remained limited, the electrical resistivity stayed within the insulating regime (~10^4^ Ω·m), the humidity sensitivity was moderate, and the structure–property relationships were primarily qualitative rather than quantitatively correlated.

Therefore, the scientific gap addressed in this work lies not merely in increasing the GO content but in systematically investigating the concentration regime where interfacial bonding, structural disorder, conductive GO domain formation, and humidity-driven proton transport intersect.

In the present study, the GO loading was increased to 3 wt.% in order to evaluate how the increased GO content affected the structural organization, electrical resistivity, and humidity-dependent conductivity in GO/CMC composite membranes [2,15,16,27]. At this GO concentration, polymer incorporation is expected to influence the inter-layer organization, interfacial interactions, electrical transport pathways, and humidity-dependent conduction behavior. Particular attention was focused on the correlation between structural modifications induced by CMC incorporation and the resulting changes in mechanical and electrical properties. Unlike many previous studies, this work establishes a quantitative structure–property–function experimental investigation by directly correlating [3,4,5,6,7,8,9,10,11,15,16,17,18,19,20,21,22,23,24,25,26,27,28,29,30,31,32]:

• FTIR band shifts and intensity variations (functional group interactions);
• XRD inter-layer spacing (d_001_) and crystallite size (Scherrer analysis);
• SEM-derived dispersion and encapsulation morphology;
• Measured tensile strength (6.6–17.8 MPa range);
• Electrical resistivity (3.43 × 10^4^ to 1.74 × 10^7^ Ω·m);
• Humidity-driven resistance variation (three to five orders of magnitude in 20–90% RH range).

The main contribution of this study is the systematic investigation of the structural, mechanical, and electrical changes occurring in 3 wt.% GO/CMC composite membranes with increasing CMC content.

Particular attention was focused on the concentration region approaching the lower percolation threshold, where interfacial interactions, inter-layer spacing variation, structural disorder, and charge transport redistribution become strongly interconnected.

To achieve this objective, FTIR, SEM, XRD, mechanical testing, four-point probe electrical measurements, and humidity-response analysis were systematically combined in order to establish correlations between the structural evolution and functional properties of the GO/CMC membranes.

**Figure 1:**
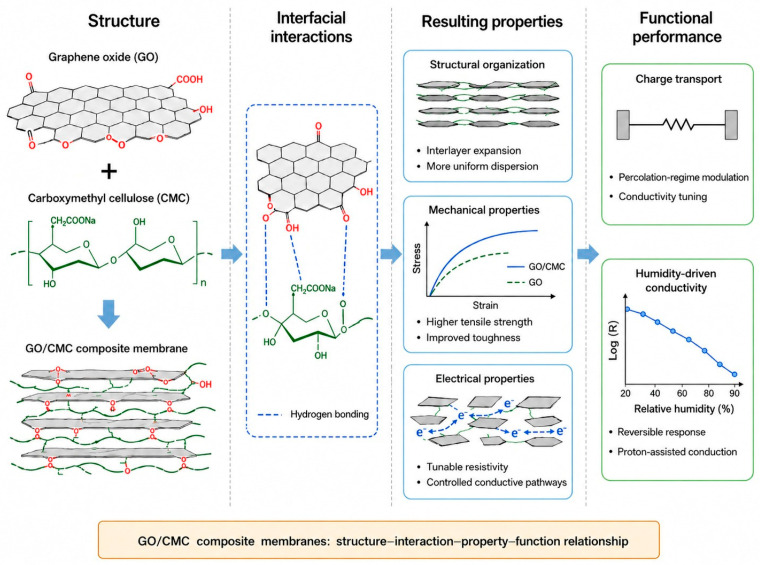
A conceptual illustration of the structure–property–function relationships in GO/CMC composite membranes, highlighting the influence of interfacial hydrogen bonding on the mechanical, electrical, and humidity-sensing properties.

The main objective of this work was to investigate the influence of the carboxymethyl cellulose content on the structural organization, mechanical behavior, electrical resistivity, and humidity-dependent conductivity of 3 wt.% graphene oxide/carboxymethyl cellulose composite membranes. Particular attention was focused on establishing correlations between the interfacial interactions, structural evolution, morphology, tensile performance, electrical resistivity, and humidity-response behavior. FTIR spectroscopy, X-ray diffraction, scanning electron microscopy, mechanical testing, electrical measurements, and humidity-response analysis were applied to evaluate the structure–property relationships within the GO/CMC composite membranes. The overall structure–property–function concept investigated in this work is illustrated schematically in Figure 1.

## 2. Experimental Section

### 2.1. Materials

All chemicals used in this study were of analytical grade and employed without additional purification. Natural graphite was obtained from the Ognevskoye graphite deposit (Ognevka mine, Ulan district, East Kazakhstan region) and used as the carbon precursor for graphene oxide synthesis.

The oxidizing reagents included potassium permanganate (KMnO_4_, 99%), sodium nitrate (NaNO_3_, 99%), and concentrated sulfuric acid (H_2_SO_4_, 98%). Additional reagents used during synthesis and purification were hydrochloric acid (HCl, 35%), hydrogen peroxide (H_2_O_2_, 31%), sodium hydroxide (NaOH, ≥99%), glacial acetic acid (CH_3_COOH, ≥85%), ethanol (C_2_H_5_OH, 96%), and trichloroacetic acid (CCl_3_COOH, 99%). All reagents were supplied by Alita LLP (Kazakhstan).

Deionized water was used throughout the synthesis and washing procedures.

In contrast to our previously reported 1 wt.% GO/CMC composite system, the present study investigates 3 wt.% GO membranes, enabling evaluation of the influence of increased GO loading on the structural organization, mechanical reinforcement, electrical transport, and humidity sensing performance.

### 2.2. Methods

#### 2.2.1. Synthesis of Graphene Oxide (GO)

Graphene oxide was synthesized from natural graphite using a modified Hummers oxidation method (Figure 1). The synthesis involved two major stages: the oxidation of graphite to graphite oxide and the subsequent exfoliation into graphene oxide sheets.

Initially, 1 g of graphite powder was placed in an ice bath maintained at approximately 0 °C. Concentrated sulfuric acid (94%) was slowly added, followed by 0.5 g NaNO_3_ and 3 g KMnO_4_, while maintaining continuous stirring. Each reagent was added sequentially with stirring intervals of approximately 15 min, followed by continuous mixing for 2 h using a magnetic stirrer.

The mixture was subsequently heated to 35 °C for 30 min to promote intermediate oxidation reactions. After the addition of deionized water, the temperature was increased to 90 °C and maintained for another 30 min, facilitating the formation of graphite oxide.

Finally, 30% hydrogen peroxide was added until the mixture turned bright yellow, indicating the completion of the oxidation process and a reduction in residual permanganate species.

The resulting suspension was filtered and washed repeatedly with 5% hydrochloric acid to remove residual metal ions. Subsequent washing with deionized water and centrifugation (5000 rpm) was performed until the pH reached ≈ 7.

**Figure 2:**
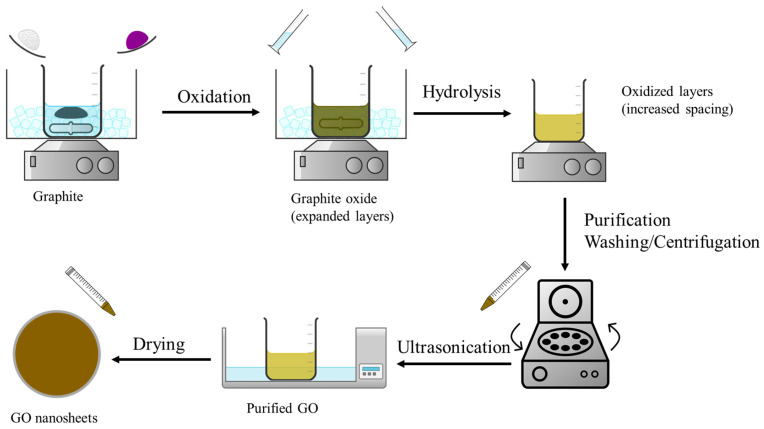
A schematic illustration of the synthesis of graphene oxide via a modified Hummers method, including the graphite oxidation, hydrolysis, purification, and ultrasonic exfoliation steps leading to the formation of graphene oxide sheets.

To achieve the exfoliation of graphite oxide into graphene oxide sheets, the suspension was subjected to ultrasonic treatment at 45 kHz for 60 min at 25 °C. This process resulted in a stable GO dispersion containing multi-layer, few-layer, and single-layer GO sheets. Graphene oxide was synthesized from natural graphite using a modified Hummers oxidation method. The overall synthesis procedure, including the oxidation of graphite and subsequent exfoliation of graphite oxide into graphene oxide sheets, is schematically illustrated in Figure 2.

#### 2.2.2. Synthesis of Carboxymethyl Cellulose (CMC)

Carboxymethyl cellulose was synthesized via the alkalization and etherification of cellulose.

Initially, 5 g of cellulose was mixed with 100 mL of 95% ethanol and 10 mL of 45% NaOH solution and stirred at 750 rpm for 60 min at room temperature. The alkaline activation of cellulose facilitated the subsequent carboxymethylation.

After activation, 5 mL of trichloroacetic acid was introduced into the mixture, and the reaction was conducted in a water bath at 60 °C for 60 min.

The resulting product was cooled to room temperature and neutralized with glacial acetic acid until a pH of 6–7 was achieved. The precipitated product was filtered and washed with 80% ethanol using a Soxhlet extractor for 3 h to remove residual reagents.

**Figure 3:**
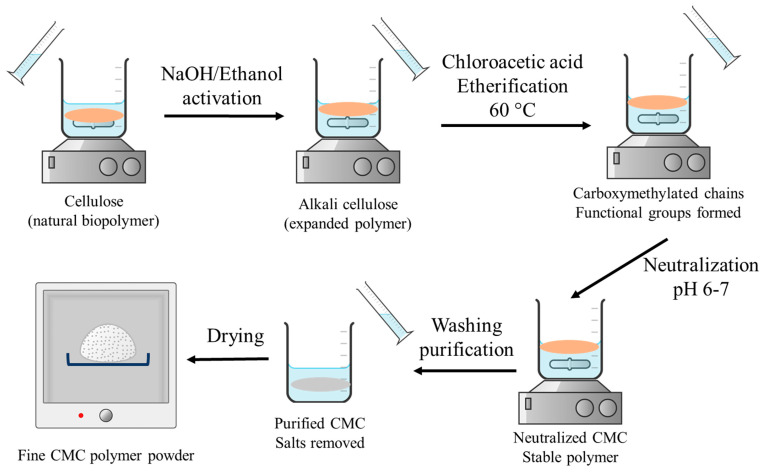
A schematic illustration of the synthesis of carboxymethyl cellulose (CMC) via the alkalization and etherification of cellulose, including the alkaline activation, etherification with trichloroacetic acid, neutralization, purification, and drying stages.

Finally, the obtained CMC powder was dried at room temperature. The synthesis of carboxymethyl cellulose involved the alkaline activation of cellulose followed by etherification with trichloroacetic acid to introduce carboxymethyl functional groups into the polymer chain. The overall synthesis route and key processing steps are presented in Figure 3.

#### 2.2.3. Preparation of GO/CMC Composite Membranes

Composite membranes were prepared by dispersing GO and CMC in aqueous solution followed by solvent casting.

Initially, a 3 wt.% GO suspension was prepared by dispersing synthesized GO in deionized water and sonicating for 30 min at 30 kHz using an ultrasonic bath.

CMC powder was separately ground using a ball mill (500 rpm, 15 min) to ensure uniform particle size.

Subsequently, CMC was gradually introduced into the GO suspension at three concentrations:

• 0.03 g;
• 0.06 g;
• 0.15 g.

The mixture was stirred using a magnetic stirrer at 800 rpm and 40 °C for 60 min, followed by ultrasonic treatment (45 kHz, 30 °C, 60 min) to ensure the homogeneous dispersion of GO sheets within the polymer matrix.

The resulting suspensions were cast onto flat polymer substrates and dried at 50 °C for 48 h, producing flexible GO/CMC composite membranes.

This approach enabled a systematic investigation of the influence of the CMC content on the properties of 3 wt.% GO composite systems, which were compared with previously reported 1 wt.% GO/CMC membranes.

**Figure 4:**
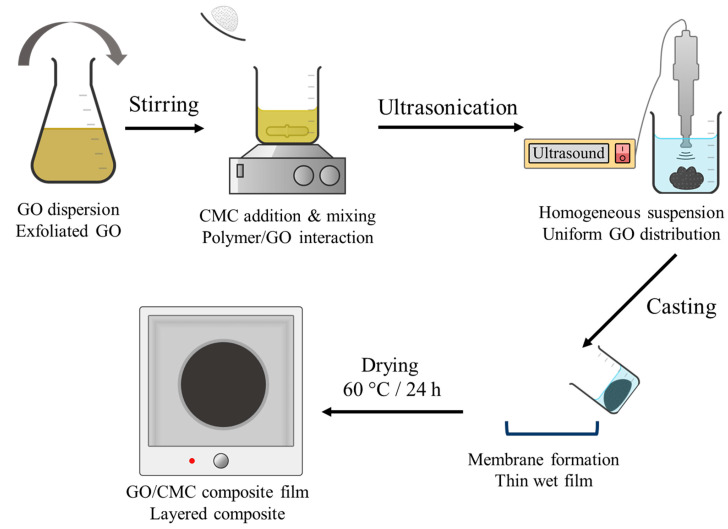
Fabrication of GO/CMC composite membranes.

The fabrication of the humidity sensor based on GO and GO/CMC composite membranes is illustrated in Figure 4. The membranes were mounted onto a dielectric substrate and connected with copper electrodes to ensure stable electrical contact during measurements.

#### 2.2.4. SEM Analysis

The surface morphology of the GO and GO/CMC composite membranes was investigated using a JEOL JSM-LV 6390 scanning electron microscope operating at an accelerating voltage of 15 kV in high-vacuum mode.

Prior to imaging, samples were coated with a thin gold layer to improve electron conductivity. The specimens were mounted on aluminum stubs using conductive carbon tape.

#### 2.2.5. FTIR Analysis

Fourier transform infrared spectroscopy (FTIR) was employed to analyze the chemical structure of GO and GO/CMC membranes.

Measurements were performed using a Simex FT-801 spectrometer in the spectral range of 450–4700 cm^−1^ with a resolution of 1 cm^−1^ at room temperature (25 °C).

The spectra were collected using a universal reflection–absorption configuration.

#### 2.2.6. X-Ray Diffraction Analysis

The crystalline structure of the GO and GO/CMC membranes was analyzed using X-ray diffraction (XRD) on an X’PertPRO diffractometer (Malvern Panalytical) with CuKα radiation (λ = 1.54187 Å).

Measurements were performed in the 2θ range of 10–40° with an X-ray tube voltage of 45 kV and current of 30 mA.

The crystallite size was estimated using the Scherrer equation:

(1) D = kλ/βcosθD

where k = 0.94 is the shape factor, λ is the X-ray wavelength, β is the full width at half maximum (FWHM), and θ is the Bragg diffraction angle.

#### 2.2.7. Mechanical Characterization

Mechanical properties of the GO and GO/CMC membranes were evaluated using a WDW-5 kN universal tensile testing machine (Time Group Inc., Beijing, China).

The measurements were conducted at a crosshead speed of 2 mm min^−1^ under a load range of 0.01–5 kN.

The tensile strength, Young’s modulus, strain at break, and toughness were calculated from the obtained stress–strain curves. The gauge length used for calculations was 10 mm.

#### 2.2.8. Electrical Characterization

The electrical resistivity of the membranes was measured using the four-point probe method to minimize contact resistance.

Current was measured using a Keithley 6485 picoammeter, while voltage was applied using a Tektronix PWS2326 power supply.

This method enabled the accurate determination of the intrinsic electrical resistivity of GO and GO/CMC membranes.

#### 2.2.9. Humidity Sensor Fabrication and Testing

Humidity sensors were fabricated by mounting GO and GO/CMC membranes onto dielectric substrates and connecting copper electrodes (0.15 mm diameter) to opposite ends of the membranes.

The membranes had the following approximate dimensions:

• Length: 3.5 cm;
• Width: 1 cm;
• Thickness: ~20 μm.

The sensors were placed in a sealed humidity-controlled chamber together with a DHT22 humidity sensor (Arduino platform) used as a reference device.

**Figure 5:**
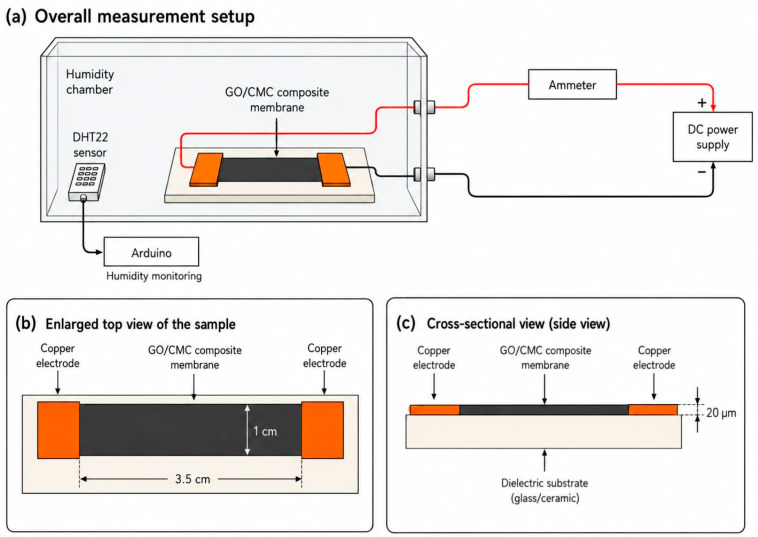
A schematic diagram of the GO/CMC humidity sensor and experimental measurement setup. (**a**) The overall measurement configuration; (**b**) the top view of the sensor; and (**c**) the cross-sectional side view of the sensing structure.

The humidity range during testing was 20–90% RH, and the electrical response was monitored using a Keithley 6485 picoammeter. The measurement error of the system did not exceed 0.5%. The fabrication of the humidity sensor based on GO and GO/CMC composite membranes is illustrated in Figure 5. The membranes were mounted onto a dielectric substrate and connected with copper electrodes to ensure stable electrical contact during measurements.

The experimental setup used to investigate the humidity sensing performance of the fabricated sensors is presented in Figure 5. The system includes a sealed humidity-controlled chamber, a reference humidity sensor, and a Keithley 6485 picoammeter used for electrical measurements.

## 3. Results and Discussion

For clarity and consistency throughout this manuscript, the investigated samples are denoted as (0) initial GO; (1) initial CMC; (2) GO/CMC 0.03 g; (3) GO/CMC 0.06 g; (4) GO/CMC 0.15 g, corresponding to composite membranes containing 3 wt.% GO with different CMC contents.

### 3.1. Fourier Transform Infrared (FTIR) Spectroscopy Analysis

**Figure 6:**
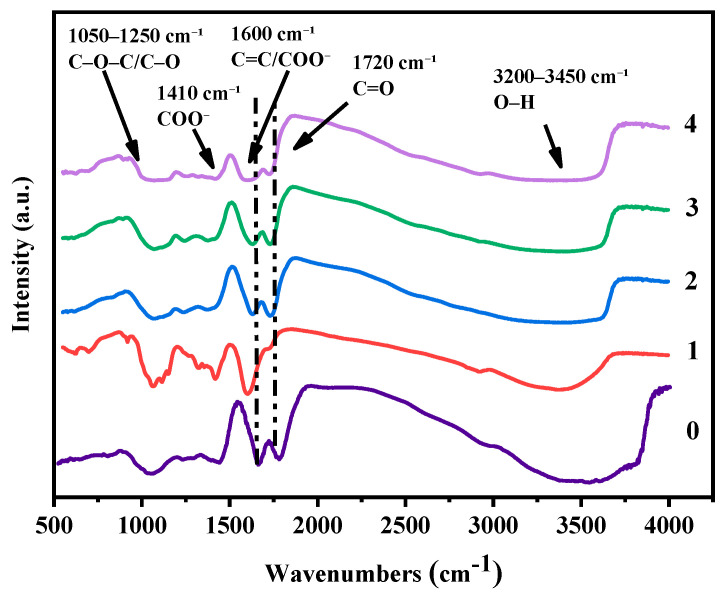
FTIR spectra of 3 wt.% GO and GO/CMC composite membranes: (0) initial GO; (1) initial CMC; (2) GO/CMC 0.03 g; (3) GO/CMC 0.06 g; (4) GO/CMC 0.15 g.

Fourier transform infrared (FTIR) spectroscopy was employed to investigate the chemical bonding and functional group interactions within the GO/CMC composite membranes. FTIR is widely recognized as a reliable method for evaluating the oxidation degree of graphene oxide, interfacial interaction mechanisms with polymer matrices, and the chemical stability of composite systems [33,34]. The corresponding spectra of the GO and GO/CMC composite membranes are presented in Figure 6.

The FTIR spectrum of pristine graphene oxide exhibits a broad and intense absorption band in the 3200–3400 cm^−1^ region, corresponding to −OH stretching vibrations associated with hydroxyl groups and adsorbed water molecules. A pronounced peak at 1720–1740 cm^−1^ is attributed to carbonyl (C=O) stretching vibrations, while the band at 1610–1630 cm^−1^ corresponds to aromatic C=C skeletal vibrations and deformation modes of inter-layer water molecules. Additionally, absorption bands in the 1050–1250 cm^−1^ range confirm the presence of epoxy (C–O–C) and alkoxy (C–O) groups. These features indicate a high oxidation degree and a surface rich in oxygen-containing functional groups, consistent with the literature data [35,36].

Notably, in the present study, the membranes containing 3 wt.% GO exhibited higher relative intensities of oxygen-related bands compared to those of previously studied 1 wt.% GO systems, indicating an increased density of functional groups per unit volume. This enhanced chemical functionality is expected to significantly influence the interfacial bonding and structural organization within the composite matrix.

In the FTIR spectra of GO/CMC composites, characteristic bands of carboxymethyl cellulose are clearly observed. The broad −OH stretching band in the 3330–3450 cm^−1^ region becomes more pronounced with increasing CMC content, suggesting increased intermolecular interactions between GO and CMC [1,27,33,34,35]. The absorption band at 1580–1600 cm^−1^ corresponds to the asymmetric stretching of carboxylate (−COO^−^) groups, while the band at 1410–1430 cm^−1^ is assigned to their symmetric stretching vibrations. Strong peaks in the 1020–1080 cm^−1^ range arise from C–O–C vibrations within the cellulose backbone.

In GO-containing composites, noticeable shifts and broadening of carbonyl and hydroxyl bands are observed, consistent with the formation of intermolecular hydrogen-bonded interactions [1,33,34] between GO sheets and CMC chains. Particularly in samples containing 0.06–0.15 g CMC, significant broadening of −OH and −COO^−^ bands suggests densification of the composite network through enhanced interfacial interactions.

These chemical modifications correlate well with the structural changes observed in the XRD analysis (inter-layer spacing variation) and the homogeneous morphology detected by SEM. Importantly, the FTIR results demonstrate that increasing the functional group density strengthens hydrogen bonding interactions, which contributes to improved mechanical strength while simultaneously restricting charge carrier mobility. Thus, the FTIR analysis supports the interpretation of intermolecular interactions within the composite membranes [33,34] for the structure–property relationships observed in GO/CMC composites and enables integrated interpretation alongside XRD and mechanical–electrical characterization.

### 3.2. Scanning Electron Microscopy (SEM) Analysis

Scanning electron microscopy (SEM) was employed to examine the morphological structure and dispersion uniformity of GO and GO/CMC composite membranes. SEM analysis is particularly valuable for evaluating the inter-layer architecture, surface porosity, and degree of interfacial interaction between polymer matrices and graphene oxide sheets, all of which play a critical role in determining mechanical and electrical performance [37,38].

**Figure 7:**
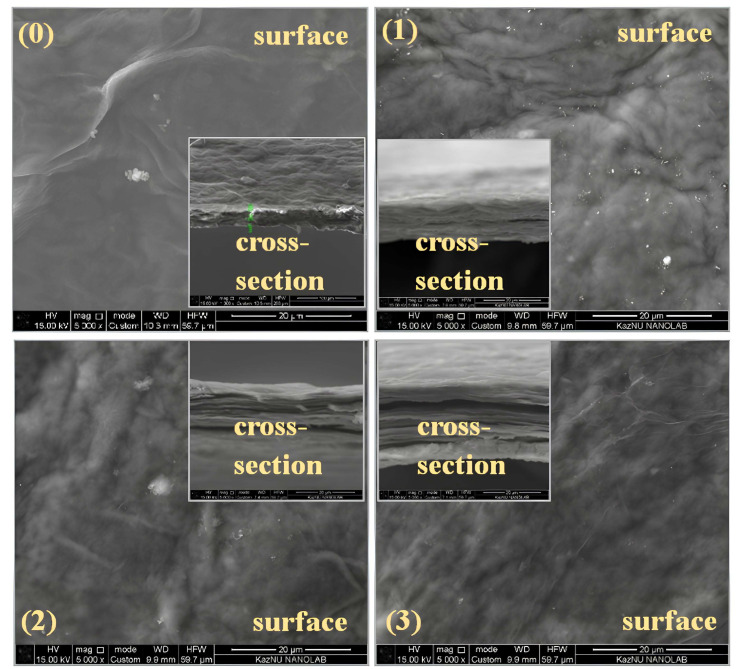
SEM images of 3 wt.% GO and GO/CMC composite membranes showing surface morphologies and cross-sectional structures: (0) initial GO; (1) GO/CMC 0.03 g; (2) GO/CMC 0.06 g; (3) GO/CMC 0.15 g.

The surface morphology and internal structure of the membranes were analyzed using scanning electron microscopy. Representative SEM images of the GO and GO/CMC composite membranes are shown in Figure 7.

SEM images of pristine GO membranes reveal a relatively smooth but distinctly layered morphology. Closely stacked GO sheets form an oriented lamellar structure, which may facilitate charge carrier transport along preferential pathways. However, weak inter-layer bonding results in limited mechanical stability and susceptibility to crack formation under tensile stress. This morphological observation is consistent with the low tensile strength value of approximately 0.1 MPa measured for pure GO membranes.

Upon incorporation of 0.03 g CMC, the morphology underwent significant modification. The polymer matrix filled the inter-layer gaps between GO sheets, forming a more uniform and compact structure. Polymer chains bridged adjacent GO layers, reducing microvoids and enhancing structural cohesion. This densification directly correlates with the hydrogen bonding interactions detected by FTIR analysis. Consequently, the mechanical strength increased to approximately 0.3 MPa, and the membrane exhibited improved deformation stability.

For GO/CMC composites containing 0.06 g CMC, SEM images reveal a highly homogeneous, continuous, and nearly pore-free morphology. GO sheets are well dispersed within the polymer matrix, and aggregation is significantly reduced. This morphology reflects effective interfacial interaction between GO and CMC. The observed structural uniformity corresponds to the increased inter-layer spacing detected by XRD and reduced crystallite size, indicating partial exfoliation and improved dispersion. As a result, the mechanical strength increases to approximately 0.5 MPa, with stable elastic–plastic behavior observed in the tensile curves.

SEM images display the densest and most structurally stable morphology for GO/CMC 0.15 g samples. The high polymer content encapsulates GO sheets, forming a continuous network structure with minimal structural defects and microvoids. This morphology correlates with the highest measured tensile strength observed for the composite membranes [3,4,5,6,7,8,9,10,11]. Morphological densification also strongly affects the electrical properties. As the CMC content increases, polymer barriers between GO sheets become thicker, interrupting conductive pathways. This structural isolation corresponds to the increase in electrical resistivity from 6.46 × 10^6^ Ω·m to 1.74 × 10^7^ Ω·m, as measured by the four-point probe method. Similar order-of-magnitude increases in resistivity due to morphological densification have been reported in GO/biopolymer systems [39,40,41].

Overall, the SEM analysis demonstrates that increasing the CMC content enhances the morphological uniformity, structural compactness, and interfacial bonding efficiency, directly correlating with improved mechanical strength and increased electrical resistivity. These findings support the observed structure–property relationship within the composite membranes [3,4,5,6,7,8,9,10,11,36] and the structure–property relationship in GO/CMC composite membranes.

### 3.3. X-Ray Diffraction (XRD) Analysis

X-ray diffraction (XRD) analysis was carried out to investigate the structural ordering, inter-layer spacing evolution, and crystallite size variation in GO and GO/CMC composite membranes. In graphene-based layered systems, XRD provides essential information on the stacking periodicity, oxidation-induced expansion, polymer intercalation, and disorder development. Since mechanical reinforcement and electrical transport in GO/polymer composites are highly sensitive to nanoscale structural organization, quantitative diffraction analysis is crucial for establishing structure–property correlations [3,4,5].

**Figure 8:**
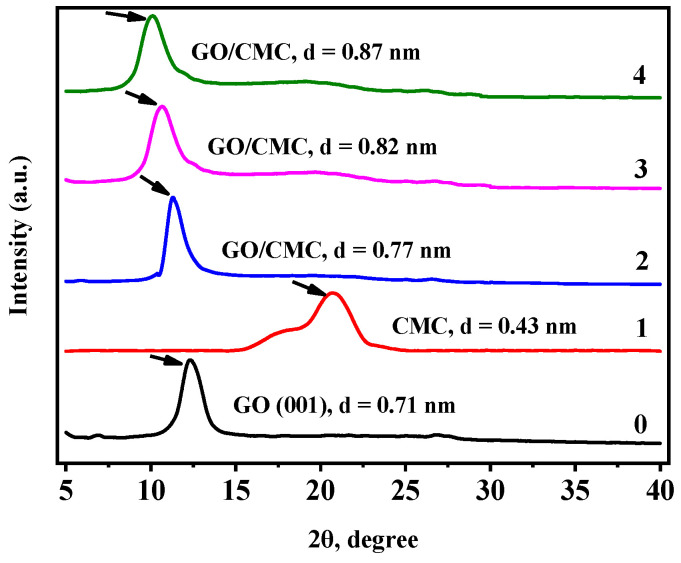
X-ray diffraction patterns of 3 wt.% GO and GO/CMC composite membranes: (0) initial GO; (1) initial CMC; (2) GO/CMC 0.03 g; (3) GO/CMC 0.06 g; (4) GO/CMC 0.15 g.

The crystalline structure and inter-layer spacing of the prepared membranes were investigated using X-ray diffraction analysis. The diffraction patterns obtained for the GO and GO/CMC composite membranes are shown in Figure 8.

Oxidation of graphite introduces oxygen-containing functional groups between graphene layers, increasing the inter-layer distance relative to pristine graphite (~0.34 nm). Upon incorporation into a polymer matrix, further structural modification may occur via intercalation, exfoliation, and encapsulation mechanisms. Therefore, analysis of the peak position, peak intensity, and peak broadening enables the systematic tracking of the structural evolution with increasing CMC content (Table 1).

X-ray diffraction analysis was used to evaluate the structural ordering, diffraction peak position, inter-layer spacing, and crystallite size of the GO and GO/CMC composite membranes. The analysis was focused on the main low-angle GO-related diffraction peak and its changes after incorporation of CMC. The inter-layer spacing was calculated using Bragg’s law, while the crystallite size was estimated using the Scherrer equation [35,38,39].

The XRD pattern of pristine GO showed a diffraction peak at 2θ = 12.41°, corresponding to an inter-layer spacing of d ≈ 0.71 nm. This value is within the commonly reported range for oxidized graphene oxide structures and indicates expansion of the graphite inter-layer distance after oxidation [35].

For the initial CMC sample, a polymer-related diffraction feature was observed at 2θ = 20.46°, corresponding to d ≈ 0.43 nm. This peak is not assigned to GO inter-layer spacing and was considered separately as a CMC-related structural contribution.

After incorporation of CMC into GO, the main GO-related diffraction peak shifted toward lower 2θ values. For GO/CMC (0.03 g), the peak appeared at 2θ = 11.48°, corresponding to d ≈ 0.77 nm. For GO/CMC (0.06 g), the peak shifted to 2θ = 10.77°, giving d ≈ 0.82 nm. For GO/CMC (0.15 g), the broadened low-angle GO-related peak was observed at 2θ = 10.21°, corresponding to d ≈ 0.87 nm.

This gradual shift toward lower diffraction angles indicates an increase in the calculated inter-layer spacing after CMC incorporation. The result suggests modification of the layered GO organization in the composite membranes. However, XRD data alone do not conclusively prove a specific intercalation mechanism. Therefore, the observed peak shift is discussed only as evidence of the structural reorganization and possible insertion of polymer chains between GO layers [35,38,39].

The crystallite size was calculated from the full width at half maximum of the diffraction peaks using the Scherrer equation. The calculated crystallite size of pristine GO was approximately 5.6 nm. For initial CMC, the crystallite size calculated from the polymer-related diffraction feature was approximately 2.5 nm. For GO/CMC (0.03 g), GO/CMC (0.06 g), and GO/CMC (0.15 g), the calculated crystallite sizes were approximately 6.6 nm, 4.3 nm, and 4.3 nm, respectively.

The decrease and broadening of diffraction features in the composite membranes indicate reduced coherent scattering lengths and the decreased long-range ordering of GO-based domains after polymer incorporation. These changes are consistent with the structural modification of the layered GO organization in the presence of CMC. However, they should not be interpreted as direct proof of stress transfer efficiency or electronic pathway formation.

Overall, the XRD results show that CMC incorporation affects the diffraction behavior of GO/CMC membranes through peak shifting, peak broadening, and changes in the calculated crystallite size. These structural changes are considered together with SEM and mechanical testing results when discussing the relationship between membrane morphology and mechanical behavior [3,4,5,6,7,8,9,10,11,15].

#### Correlation Between Structural, Mechanical, and Electrical Behavior

The XRD results obtained for the GO and GO/CMC composite membranes demonstrate that incorporation of CMC influences the layered structural organization of graphene oxide and modifies the diffraction behavior of the composite system. These structural changes are also reflected in the FTIR spectra, SEM morphology, mechanical properties, and electrical response of the membranes.

The shift in the main GO-related diffraction peak toward lower 2θ values after addition of CMC indicates an increase in the calculated inter-layer spacing from approximately 0.71 nm for pristine GO to approximately 0.77–0.87 nm for the GO/CMC composites. Simultaneously, peak broadening becomes more pronounced at higher CMC contents. In layered graphene oxide systems, such broadening is commonly associated with reduced long-range ordering, increased structural heterogeneity, and the partial disruption of the regular stacking periodicity [35,38,39].

The FTIR spectra support this interpretation. In particular, broadening of the −OH stretching region together with changes in the −COO^−^ vibration bands indicates intermolecular interactions between oxygen-containing functional groups of GO and hydroxyl/carboxyl groups of CMC. These spectral changes are consistent with the structural modifications observed by XRD and suggest that polymer incorporation affects the arrangement of GO layers within the composite membrane [35,38,39].

The SEM observations are also consistent with the diffraction analysis. Pristine GO membranes exhibited a layered morphology with the relatively compact stacking of GO sheets. After addition of CMC, the membrane morphology became more homogeneous and compact, while visible aggregation decreased. For the GO/CMC (0.15 g) sample, SEM images show a denser polymer-rich structure with fewer visible pores and more continuous surface coverage. These observations correlate with the attenuation and broadening of the GO-related diffraction peak in XRD patterns.

The structural changes identified by XRD and SEM are reflected in the mechanical properties of the membranes. Tensile testing showed that the tensile strength increased from approximately 6.6 MPa for pristine GO to 17.8 MPa for GO/CMC (0.15 g). The Young’s modulus also increased with the increasing CMC content. The mechanical improvement may be associated with increased intermolecular interactions between GO sheets and CMC chains together with more homogeneous structural organization within the membrane [3,4,5,6,7,8,9,10,11,15].

At the same time, the XRD results indicate that increasing the polymer content modifies the layered GO arrangement and increases the average inter-layer spacing. Such structural modification can influence electron transport between adjacent GO domains. The electrical measurements showed that the dry-state resistivity increased progressively from approximately 5.8 × 10^6^ Ω·m for pristine GO to 2.32 × 10^7^ Ω·m for GO/CMC (0.15 g). This increase in resistivity is consistent with the increasing polymer contribution within the composite structure and reduced electronic coupling between GO domains.

The combined XRD and electrical measurements suggest that the polymer phase acts as a partially insulating component separating adjacent GO sheets. As the average inter-layer distance increases and diffraction peaks broaden, long-range structural ordering decreases, which may contribute to the reduced continuity of charge transport pathways within the membrane [3,4,5,6,7,8,9,10,11,15]. However, the present XRD data alone do not directly prove a specific electrical transport mechanism, and therefore the electrical behavior is interpreted only in correlation with the structural observations.

The humidity-response behavior of the membranes also correlates with the structural evolution observed by XRD. Although the dry-state resistivity increased with the increasing CMC content, all samples showed a significant decrease in resistance at elevated humidity levels. This behavior is consistent with the adsorption of water molecules within the hydrophilic GO/CMC structure and the formation of proton conduction pathways in hydrated regions of the membrane.

The increased inter-layer spacing observed in the GO/CMC composites may facilitate the penetration and retention of water molecules between GO-containing domains. At higher humidity, adsorbed water layers can contribute to proton-assisted conduction processes, leading to a decrease in electrical resistance. The GO/CMC (0.15 g) membrane exhibited the highest dry-state resistivity but still maintained a pronounced humidity-dependent electrical response, indicating that water adsorption remains active despite increased polymer content.

The XRD results therefore provide structural information that complements the FTIR, SEM, mechanical, and electrical analyses. The diffraction peak shift, peak broadening, and changes in the calculated crystallite size collectively indicate the gradual modification of the layered GO organization after incorporation of CMC. These structural changes are consistent with the observed improvement in mechanical stability, the increase in dry-state electrical resistivity, and the humidity-dependent electrophysical behavior of the GO/CMC composite membranes.

Overall, the combined analysis suggests that the functional properties of the membranes are governed by the balance between the structural ordering of GO domains, polymer-related intermolecular interactions, and the resulting modification of mechanical and electrical behavior within the composite system [3,4,5,6,7,8,9,10,11,15].

### 3.4. Mechanical Characterization

The mechanical performance of the 3 wt.% GO membrane and GO/CMC composite membranes was systematically investigated to evaluate the structural reinforcement mechanisms induced by polymer incorporation (Table 2). In graphene oxide-based layered systems, tensile behavior is governed by the inter-layer bonding strength, load transfer efficiency, and microstructural homogeneity [10,11,15]. The incorporation of carboxymethyl cellulose (CMC) modifies interfacial interactions through hydrogen bonding and polymer intercalation, thereby altering the stiffness, tensile strength, and fracture resistance [28,29].

**Figure 9:**
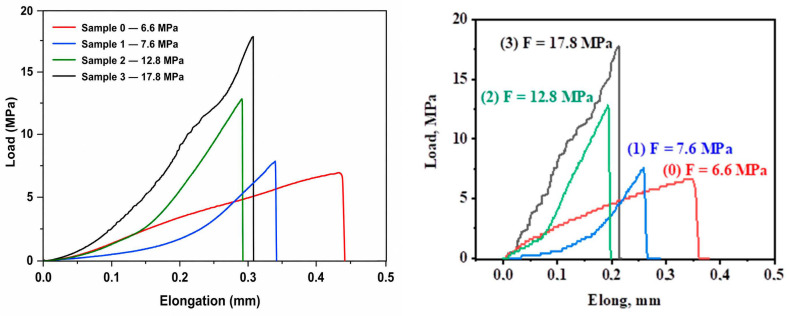
Stress–strain curves of 3 wt.% GO and GO/CMC composite membranes: (0) initial GO; (1) GO/CMC 0.03 g; (2) GO/CMC 0.06 g; (3) GO/CMC 0.15 g.

To ensure a quantitative comparison, the tensile strength, Young’s modulus (calculated from the initial linear elastic region), strain at break, and mechanical energy absorption (toughness) were determined from the stress–strain curves. The mechanical properties of the prepared membranes were evaluated by tensile testing. The stress–strain curves obtained for the GO and GO/CMC composite membranes are presented in Figure 9.

#### 3.4.1. Mechanical Performance Parameters

Young’s modulus was calculated using the linear regression of the elastic region:

(2) E = dε/dσ

The mechanical energy absorption (toughness, U) was determined from the area under the stress–strain curve assuming quasi-linear deformation:

(3) U = 1/2σ_max_ε_break_

#### 3.4.2. Mechanical Behavior of Pristine GO Membrane

The pristine 3 wt.% GO membrane exhibits a tensile strength of 6.6 MPa and Young’s modulus of 314 MPa. The stress–strain curve shows relatively brittle behavior with limited deformation prior to fracture, with a strain at break of approximately 2.9%.

This mechanical response originates from the layered structure of GO identified by XRD analysis (inter-layer spacing ≈ 0.71 nm), where adjacent sheets are predominantly held together by weak van der Waals interactions [10,11]. SEM observations reveal a lamellar stacking morphology, which promotes inter-layer slippage under tensile loading and facilitates crack propagation [15].

Nevertheless, the obtained tensile strength is significantly higher than that reported for many pure GO films due to the relatively dense structure formed at higher GO loading. The increased oxide content results in a more interconnected network of GO sheets, contributing to improved mechanical integrity [28].

#### 3.4.3. Mechanical Properties of GO/CMC (0.03 g) Composite

The incorporation of 0.03 g CMC led to an increase in tensile strength to 7.6 MPa, while the strain at break increased to 3.79%, indicating the improved ductility of the composite membrane.

FTIR analysis confirms the formation of hydrogen bonding between hydroxyl groups of GO and carboxyl groups of CMC, enhancing interfacial adhesion within the composite matrix [28,29]. SEM images show a homogeneous dispersion of GO sheets within the polymer matrix, enabling more uniform stress distribution during tensile loading.

Interestingly, the Young’s modulus decreases to 126 MPa, indicating that the addition of a small amount of polymer introduces flexibility into the composite system. This reduction in stiffness combined with increased elongation suggests improved toughness, which increases to 0.252 MJ/m^3^.

Such polymer-induced ductility is commonly observed in GO/polymer composites where the polymer phase partially relaxes the rigid layered structure of graphene oxide [29,30,31].

#### 3.4.4. Mechanical Behavior of GO/CMC (0.06 g) Composite

At a CMC content of 0.06 g, the composite exhibits significant improvements in the tensile strength to 12.8 MPa and in the Young’s modulus to 568 MPa.

XRD analysis reveals increased inter-layer spacing (≈0.85 nm) and a reduced crystallite size, indicating partial structural disorder and effective polymer intercalation between GO layers [11,28]. These structural changes promote improved stress transfer between adjacent GO sheets.

The stress–strain curve shows a well-defined elastic region followed by gradual fracture, indicating improved load-bearing capacity. The toughness increases to 0.295 MJ/m^3^, reflecting improved energy absorption capability.

Such improvements are consistent with previously reported GO/cellulose-based composites, where polymer incorporation may contribute to the improved mechanical behavior of the composite membranes [3,4,5,6,7,8,9,10,11].

#### 3.4.5. High Strength of GO/CMC (0.15 g) Composite

The highest mechanical performance was achieved at 0.15 g CMC content, where the tensile strength reached 17.8 MPa, the Young’s modulus reached 838 MPa, and the toughness reached 0.512 MJ/m^3^.

SEM observations reveal the nearly complete encapsulation of GO sheets within the polymer matrix, minimizing structural defects and microvoid formation [29]. XRD patterns indicate attenuation of the characteristic GO peak and the increasing dominance of polymer-associated diffraction features, suggesting a transition toward a polymer-dominated network structure [28].

The formation of an interconnected polymer-rich structure may improve stress redistribution within the membrane [3,4,5,6,7,8,9,10,11]. As a result, both the stiffness and fracture resistance increase simultaneously, leading to a substantial improvement in the mechanical stability [30,31].

#### 3.4.6. Comparison Between 1 wt.% and 3 wt.% GO Systems

To better understand the influence of the GO concentration, the obtained mechanical properties were compared with those of our previously reported 1 wt.% GO/CMC composite membranes (Table 3) [27].

The comparison clearly shows that increasing the GO concentration from 1 wt.% to 3 wt.% significantly enhances the stiffness of the composite system while also improving the tensile strength.

Higher GO loading results in:

• Increased density of reinforcing nanosheets;
• Improved stress transfer pathways;
• Formation of a more rigid layered network structure.

At the same time, polymer incorporation maintains sufficient flexibility and prevents brittle fracture.

#### 3.4.7. Correlation Between Mechanical and Electrical Properties

The improvement in mechanical strength occurs concurrently with changes in electrical behavior. As the CMC content increases, structural cohesion and stiffness improve; however, the polymer phase also increases the separation between conductive GO domains.

As previously discussed in Section 3.5, the electrical resistivity increased from approximately 10^4^ Ω·m to 10^7^ Ω·m with the increasing polymer content. This behavior reflects the classical trade-off between mechanical reinforcement and electrical conductivity in GO/biopolymer composites [16,32].

Hydrogen-bond-mediated reinforcement enhances the mechanical stability while simultaneously increasing the tunneling distance between conductive GO domains, thereby reducing inter-layer electronic coupling [32]. Such mechanical–electrical coupling effects are characteristic of percolative composite systems near the conductivity threshold [16].

##### Concluding Remarks for Section 3.4

Mechanical analysis demonstrates that increasing the CMC content in GO/CMC composites induces significant structural reorganization, strengthens interfacial adhesion, and enhances the load transfer efficiency.

The tensile strength increased from 6.6 MPa to 17.8 MPa, the Young’s modulus increased from 314 MPa to 838 MPa, and the toughness increased nearly fivefold.

These improvements are fully consistent with the FTIR, SEM, and XRD results, confirming a strong structure–property correlation [10,11,15,28,29,30,31]. The results further demonstrate that increasing the GO loading from 1 wt.% to 3 wt.% significantly enhances the mechanical stiffness and load-bearing capability.

The tunable mechanical–electrical coupling observed in the GO/CMC membranes highlights their strong potential for applications in flexible electronics, humidity-sensing systems, and multifunctional protective coatings.

### 3.5. Electrical Characterization

**Figure 10:**
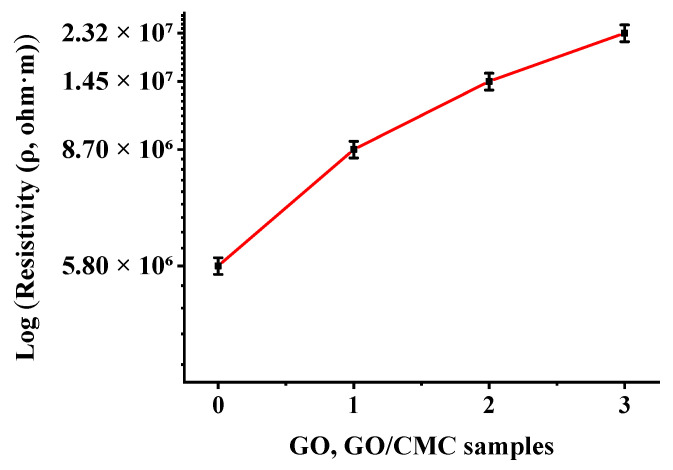
Electrical response of humidity sensors based on 3 wt.% GO and GO/CMC composite membranes: (0) initial GO; (1) GO/CMC 0.03 g; (2) GO/CMC 0.06 g; (3) GO/CMC 0.15 g.

The electrical properties of the GO and GO/CMC composite membranes were systematically investigated to evaluate the intrinsic charge transport behavior and to establish correlations with the structural evolution and mechanical reinforcement. Since graphene oxide is a partially oxidized, electronically disrupted carbon material, its electrical response is governed by the oxygen-containing functional groups, inter-layer spacing, defect density, and percolation network continuity within the polymer matrix [17,18,19]. The electrical characteristics of the membranes under different humidity conditions were investigated by monitoring their resistance changes. The obtained electrical response curves are presented in Figure 10.

In this study, membranes containing 3 wt.% GO and varying CMC contents (0.03 g, 0.06 g, and 0.15 g) were analyzed and directly compared with our previously reported 1 wt.% GO/CMC system [27] in order to clarify the effect of increased GO loading on electronic transport near the lower percolation regime.

All measurements discussed in this section were performed under dry conditions (20% RH) to isolate intrinsic electronic conduction mechanisms from humidity-induced proton transport (discussed separately in Section 3.6).

#### 3.5.1. Measurement Method and Electrical Properties of Pristine GO

The bulk resistivity (ρ) was determined using a four-point probe configuration connected to a high-sensitivity picoammeter (Keithley Model 6485). This method eliminates contact resistance artifacts and ensures the accurate determination of the intrinsic resistivity. Measurements were conducted under controlled ambient conditions (20% RH) for all samples.

The obtained dry-state resistivity values for pristine GO and GO/CMC composites are summarized in Table 4.

According to Table 4, pristine GO at 3 wt.% exhibits significantly higher resistivity (5.8 × 10^6^ Ω·m) compared with the 1 wt.% GO (≈1.51 × 10^6^ Ω·m) reported in the literature [27]. The approximately three- to fourfold increase reflects the impact of greater oxide content, enhanced structural disorder, and suppressed π–π stacking continuity on electronic transport pathways.

Unlike reduced graphene systems, increasing the GO concentration does not necessarily enhance conductivity. Instead, the higher density of oxygen functional groups disrupts conjugated sp^2^ domains, limiting long-range electron mobility. XRD analysis confirmed increased inter-layer spacing, while Scherrer analysis revealed a reduced crystallite size, both contributing to diminished electronic coupling.

As a result, long-range electronic transport is suppressed due to the reduced overlap between sp^2^ domains. The 3 wt.% GO membrane exhibits electrical behavior consistent with increased structural disorder and partial disruption of conductive pathways [15,16,17,28,29,30,31,32].

#### 3.5.2. Electrical Properties of GO/CMC (0.03 g) Composite

Incorporation of 0.03 g CMC into the 3 wt.% GO matrix increased the resistivity to ρ = 8.7 × 10^6^ Ω·m. This represents an approximately 50% increase relative to pristine 3 wt.% GO. The resistivity increase arises from several interrelated mechanisms:

1. FTIR spectra suggest enhanced intermolecular interactions between GO and CMC functional groups. In particular, the broadening and partial shifting of the −OH stretching band together with changes in the −COO^−^ vibration region indicate the formation of hydrogen-bonded interactions between graphene oxide sheets and CMC chains [1,27,33,34]. Although FTIR does not directly quantify the hydrogen bond strength, the observed spectral changes support the presence of stronger intermolecular interactions and improved interfacial compatibility between GO and CMC.
2. Increased insulating polymer contribution: CMC acts as an insulating matrix, increasing interdomain separation and forcing charge carriers to traverse longer tunneling distances [2,15,16].
3. Interfacial Polarization Effects: Heterogeneous GO–CMC interfaces enhance charge accumulation (Maxwell–Wagner effect), modifying conduction behavior.

A similar qualitative trend was reported for the 1 wt.% GO/CMC system [27], where addition of 0.03 g CMC increased the resistivity from 1.51 × 10^6^ to 6.46 × 10^6^ Ω·m. However, the absolute resistivity values of the 3 wt.% system remain higher, indicating stronger dielectric separation and a more pronounced disruption of electronic pathways at elevated GO loading.

#### 3.5.3. Effect of Increasing CMC Content (0.06–0.15 g)

Further increase in the CMC concentration resulted in a systematic and monotonic rise in resistivity:

• GO/CMC (0.06 g):

ρ = 1.45 × 10^7^ Ω·m;

• GO/CMC (0.15 g):

ρ = 2.32 × 10^7^ Ω·m.

The progressive increase confirms the controlled modulation of electronic transport as the polymer fraction increases. Several structural factors contribute to this behavior:

1. Hydrogen Bond Formation: Stronger intermolecular hydrogen bonding enhances interfacial adhesion but reduces direct electronic overlap between adjacent GO sheets.
2. Dielectric Encapsulation: CMC creates insulating barriers around GO domains, increasing effective tunneling distances and reducing hopping probability.
3. Domain Fragmentation (XRD–Scherrer Correlation): As shown in Section 3.3, the crystallite size decreased from ~31 nm to 2–4 nm with the increasing CMC content. This refinement increases boundary scattering and interrupts continuous conduction pathways.
4. Increased Structural Disorder: Inter-layer expansion (0.71 nm → 0.85 nm) further weakens electronic coupling.

In the 1 wt.% GO/CMC membranes [27], the resistivity increased to approximately 1.26 × 10^7^ Ω·m at 0.15 g CMC. In contrast, the 3 wt.% GO composites reached 2.32 × 10^7^ Ω·m under an identical polymer content. This comparison demonstrates that higher GO loading enhances dielectric dominance and shifts the system closer to the lower percolation threshold.

Importantly, the observed resistivity increase does not indicate collapse of the conductive network; rather, it reflects a modification of charge transport pathways within the composite membrane [15,16,17,28,29,30,31,32] governed by structural evolution.

#### 3.5.4. Correlation Between Electrical and Mechanical Properties

The evolution of the electrical resistivity correlates directly with the mechanical reinforcement described in Section 3.4.

With the increasing CMC content:

• The tensile strength increased from 6.6 MPa (pristine GO) to 17.8 MPa (0.15 g CMC);
• The resistivity simultaneously increased from 5.8 × 10^6^ to 2.32 × 10^7^ Ω·m.

This behavior represents a classical yet tunable mechanical–electrical trade-off:

• Enhanced hydrogen bonding improves the stress transfer efficiency;
• Improved interfacial adhesion strengthens the composite.

However, increased dielectric separation suppresses electronic conduction.

Compared with the 1 wt.% GO system [27], 3 wt.% GO membranes achieve higher mechanical robustness while maintaining predictable electrical tunability. This indicates that the GO concentration governs both the stress distribution and electrical transport influenced by the structural organization and conductive domain distribution [15,16,17,28,29,30,31,32].

Thus, structural disorder (XRD), domain refinement (Scherrer), hydrogen bonding (FTIR), and morphological encapsulation (SEM) collectively regulate mechanical reinforcement and electrical modulation within this unified structure–property experimental investigation.

##### Concluding Statement for Section 3.5

The electrical properties of GO/CMC composite membranes can be widely tuned by adjusting the CMC content at 3 wt.% GO loading. The systematic increase in the dry-state resistivity from 5.8 × 10^6^ to 2.32 × 10^7^ Ω·m demonstrates the controlled modulation of electronic pathways near the lower percolation regime.

The observed electrical behavior is fully consistent with the structural evolution identified by XRD (inter-layer expansion and crystallite refinement), SEM (morphological densification and polymer encapsulation), and FTIR (enhanced hydrogen bonding).

This integrated analysis establishes a comprehensive structure–property–function correlation experimental investigation. The tunable mechanical–electrical coupling enhances the applicability of the developed membranes in multifunctional flexible electronic devices, dielectric films, humidity-responsive systems, and sustainable sensing technologies.

### 3.6. Electrophysical Characteristics of Humidity Sensor Based on 3 wt.% GO and GO/CMC (0.03 g; 0.06 g; 0.15 g)

**Figure 11:**
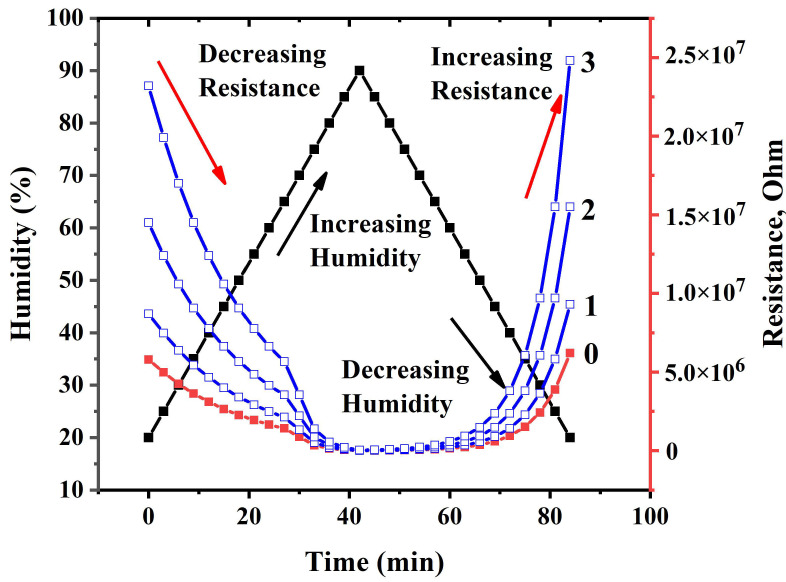
Humidity-sensing characteristics of sensors based on 3 wt.% GO and GO/CMC composite membranes: (0) initial GO; (1) GO/CMC 0.03 g; (2) GO/CMC 0.06 g; (3) GO/CMC 0.15 g.

The electrophysical behavior of humidity sensors fabricated from 3 wt.% graphene oxide (GO) and GO/carboxymethyl cellulose (CMC) composite membranes was systematically investigated in the relative humidity (RH) range of 20–90%. Measurements were conducted under dynamic humidity cycling (response–recovery mode), enabling evaluation of the sensitivity, reversibility, conduction mechanisms, and structure–property correlations as a function of the CMC content. In contrast to the previously reported 1 wt.% GO/CMC system [27], the present 3 wt.% GO configuration provides a shifted resistance window, improved structural stiffness, and a more pronounced coupling between dry-state electrical resistivity and humidity-driven proton transport. The humidity sensing performance of the fabricated sensors was evaluated in the relative humidity range of 20–90% RH. The dynamic response and recovery behavior of the sensors are shown in Figure 11.

#### 3.6.1. Humidity-Dependent Resistance Behavior

All investigated membranes exhibited a pronounced and reversible dependence of electrical resistivity on relative humidity. As the RH increased from 20% to 90%, the resistivity decreased significantly; during the recovery cycle (90% → 20% RH), the resistivity values increased correspondingly, indicating that the adsorption and desorption processes were predominantly physisorption-controlled, as commonly reported in GO-based humidity sensors [22,23,24].

For pristine 3 wt.% GO, the resistance decreased from 5.8 × 10^6^ Ω·m (20%RH) to 8.67 × 10^3^ Ω·m (90%RH), representing a nearly three orders of magnitude dynamic range. This response profile is consistent with proton-assisted conduction mechanisms in hydrated GO systems, where adsorbed water molecules facilitate ionic transport via Grotthuss-type proton hopping [25,26,42].

With the increasing CMC content, the dry-state resistivity (at 20% RH) increased progressively:

• GO/CMC (0.03 g): 8.7 × 10^6^ Ω·m;
• GO/CMC (0.06 g): 1.45 × 10^7^ Ω·m;
• GO/CMC (0.15 g): 2.32 × 10^7^ Ω·m.

At high humidity (90% RH), however, all samples converged into the low-resistance regime:

• GO: 8.67 × 10^3^ Ω·m;
• GO/CMC (0.03 g): 1.30 × 10^4^ Ω·m;
• GO/CMC (0.06 g): 2.17 × 10^4^ Ω·m;
• GO/CMC (0.15 g): 3.47 × 10^4^ Ω·m.

This convergence indicates that at elevated humidity levels, intrinsic electronic transport through the GO experimental investigation becomes secondary to water-assisted proton conduction. The observed resistance decrease at elevated humidity is consistent with previously reported proton-assisted conduction mechanisms in GO-based membranes [20,22,23]. The formation of continuous hydrogen-bonded water networks enables Grotthuss-type proton hopping, significantly increasing conductivity [25,26,42].

Compared with our earlier 1 wt.% GO/CMC system [27], the present 3 wt.% GO membranes exhibited a noticeably higher dry-state resistance window, shifting from the order of 10^6^ Ω·m in the 1 wt.% system to the order of 10^6^–10^7^ Ω·m in the 3 wt.% system. At the same time, the high-humidity resistance remained within the low-resistance regime, indicating that the humidity-induced proton conduction mechanism remained dominant despite the increase in GO loading. Thus, the main effect of increasing the GO concentration from 1 wt.% to 3 wt.% is not the suppression of humidity response but rather the controlled upward shift in the operating resistance window.

#### 3.6.2. Effect of CMC Content on Electrical Properties

The progressive increase in the dry-state resistance with the increasing CMC content aligns with dielectric encapsulation effects described in GO/polymer composites near percolation thresholds [17,18,19,20,21,22]. Several structural mechanisms contribute:

1. Hydrogen Bond Formation

Strong intermolecular hydrogen bonding between −OH and −COO^−^ groups of GO and CMC disrupts the π–π stacking continuity and increases intersheet separation, as indicated by FTIR [24,25,26,42].

2. Increased insulating polymer contribution

The insulating polymer matrix increases the interdomain tunneling distance between GO sheets and suppresses electronic pathways, consistent with increased resistivity values.

3. Interfacial Polarization (Maxwell–Wagner Effect)

Heterogeneous GO–CMC interfaces enhance the charge accumulation at phase boundaries, modifying low-frequency conduction behavior.

4. Domain Fragmentation (XRD–Scherrer Correlation)

As demonstrated in Section 3.3, increasing the CMC content reduced the crystallite size from ~31 nm to 2–4 nm and promoted structural disorder. This domain refinement increases boundary scattering and contributes to higher resistance under dry conditions.

Simultaneously, enhanced humidity sensitivity confirms that hydrophilic polymer incorporation amplifies water adsorption and proton mobility pathways [24,25,26,42]. Thus, CMC plays a dual role: it is electrically insulating under dry conditions while promoting proton conductivity at high humidity.

These structural factors have also been discussed qualitatively in previous GO/CMC humidity studies [22,23,24,27] but are here quantitatively correlated with electrical response for the first time in a 3 wt.% GO system. Notably, despite the increase in dry resistivity, the humidity sensitivity remains stable, indicating that proton conduction via adsorbed water layers dominates in the high-RH regime.

In comparison with the 1 wt.% GO/CMC system [27], the present 3 wt.% GO composites consistently exhibits higher dry-state resistivity for all CMC concentrations. For example, at 0.15 g CMC, the resistivity increases from approximately 1.26 × 10^7^ Ω·m in the 1 wt.% system to 2.32 × 10^7^ Ω·m in the 3 wt.% system. This difference confirms that increasing GO loading enhances dielectric dominance and shifts the composite closer to the lower percolation boundary while maintaining strong humidity responsiveness.

#### 3.6.3. Mechanism of Humidity Sensing

The humidity-response mechanism can be divided into three regimes:

1. Low-Humidity Region (20–40% RH)

Water adsorption is limited to isolated molecular layers. Charge transport is governed primarily by:

• Electron hopping between localized GO domains;
• Defect-mediated conduction;
• Minimal proton mobility.

Resistance remains high due to limited hydrogen-bonded network formation.

2. Intermediate-Humidity Region (40–70% RH)

Multi-layer adsorption occurs. Hydrogen-bonded networks form between GO functional groups and CMC chains. Proton hopping via the Grotthuss mechanism becomes significant, accelerating conductivity [25,26,42]. This accelerates ionic conduction and reduces resistivity.

3. High-Humidity Region (70–90% RH)

At high humidity, a quasi-continuous water film develops within expanded GO inter-layers and along hydrophilic CMC chains. Proton conduction dominates, and ionic pathways become continuous, resulting in a sharp resistivity decrease. Convergence of resistivity values near 90% RH across all samples confirms that water-mediated proton transport outweighs structural differences at high moisture levels.

These observations are generally consistent with previously reported GO humidity-sensing behavior [20,22,23]. Compared with the previously reported 1 wt.% GO system [27], the present 3 wt.% GO membranes operate in a higher baseline resistance regime but preserve the same proton-assisted sensing mechanism. This indicates that increased GO loading modifies the dry-state electrical window more strongly than the moisture-induced transport pathway itself.

#### 3.6.4. Recovery and Reversibility

During recovery (90% → 20% RH), the resistivity increased symmetrically for all samples, indicating reversible adsorption–desorption behavior consistent with physical water molecule interactions rather than irreversible chemical modification. Representative recovery values are:

• GO: 8.67 × 10^3^ → 5.8 × 10^6^ Ω·m;
• GO/CMC (0.03 g): 1.30 × 10^4^ → 8.7 × 10^6^ Ω·m;
• GO/CMC (0.06 g): 2.17 × 10^4^ → 1.45 × 10^7^ Ω·m;
• GO/CMC (0.15 g): 3.47 × 10^4^ → 2.32 × 10^7^ Ω·m.

These values reflect near-identical forward and reverse trajectories, confirming reproducible humidity response.

In comparison with the 1 wt.% GO/CMC system [27], the 3 wt.% GO membranes show the same qualitative reversibility trend but with a clearly shifted resistance scale. This confirms that the higher GO concentration does not compromise response reversibility; instead, it broadens the operating resistance range.

#### 3.6.5. Quantitative Sensitivity Enhancement

The humidity sensitivity factor was evaluated as S = R_20%RH_/R_90%RH_.

For all compositions:

• GO ≈ 6.7 × 10^2^;
• GO/CMC (0.03 g) ≈ 6.7 × 10^2^;
• GO/CMC (0.06 g) ≈ 6.7 × 10^2^;
• GO/CMC (0.15 g) ≈ 6.7 × 10^2^.

This relatively constant response ratio indicates that while the CMC content modulates the baseline resistivity, the relative humidity sensitivity remains stable, contrasting with the earlier 1 wt.% GO/CMC results reported in [27], where the baseline resistance was lower and the percolative conduction window was narrower.

Therefore, the present 3 wt.% GO system does not necessarily amplify the formal sensitivity factor but rather provides a broader, more tunable, and mechanically more stable operating resistance range for humidity-sensing applications. In this sense, the improvement is not simply in the magnitude of the S but in the integration of higher dry-state resistivity, stronger structural stability, and preserved humidity response.

#### 3.6.6. Structure–Property–Function Correlation

The electrophysical characteristics directly correlate with the structural findings:

• XRD peak shifts indicate increased inter-layer spacing, facilitating water diffusion into the GO matrix.
• Reduced crystallite size (Scherrer analysis) enhances the active interfacial area for water–GO/CMC interaction.
• SEM morphology confirms a homogeneous, pore-free structure with effective polymer encapsulation.
• FTIR analysis verifies strong hydrogen bonding interactions between GO and CMC functional groups.

Together, structural disorder, domain fragmentation, hydrogen bonding, and morphological uniformity govern both the mechanical reinforcement and humidity-driven electrical response. The increase in the dry-state resistivity with the CMC addition reflects modification of the electrical transport behavior due to structural changes and water adsorption [20,22,23,24,25,26], while the robust humidity response arises from water-assisted proton conduction pathways within hydrated GO-containing domains [20,22,23,24,25,26,42]. This dual behavior highlights the tunable mechanical–electrical–humidity coupling achieved at 3 wt.% GO loading.

Compared with the 1 wt.% GO system [27], the present 3 wt.% GO membranes show a stronger coupling between structural densification, resistivity modulation, and humidity response because the higher GO loading increases the contribution of sheet-to-sheet electronic decoupling while still preserving proton-mediated conduction under humid conditions.

#### 3.6.7. Performance Optimization and Practical Implications

Among all samples, GO/CMC (0.15 g) exhibited the highest dry-state resistance while maintaining stable humidity sensitivity. Although elevated resistivity may limit certain low-power electronic applications, the combination of the following makes these materials promising:

• Higher mechanical stiffness;
• A wide dynamic response range;
• Reversible behavior;
• Improved structural stability.

Moreover, the tunable electrical window makes these materials promising for:

• High-sensitivity humidity sensors;
• Environmental monitoring systems;
• Flexible and wearable electronics;
• Moisture-responsive functional coatings;
• Smart packaging applications.

Balancing the CMC content allows for the controlled modulation of the mechanical robustness, baseline resistivity, and humidity sensitivity to meet specific device requirements.

Relative to the 1 wt.% GO system, the 3 wt.% GO membranes offer a more robust composite membrane system, combining improved mechanical performance and higher electrical tunability while preserving humidity responsiveness.

#### 3.6.8. Conclusions of Section 3.6

The 3 wt.% GO/CMC composite membranes exhibit tunable electrophysical properties governed by structural refinement and interfacial interactions. Increasing the CMC concentration shifted the dry-state resistivity from 5.8 × 10^6^ to 2.32 × 10^7^ Ω·m while preserving a stable humidity sensitivity factor of ~6.7 × 10^2^ across all compositions.

The integration of XRD, Scherrer, FTIR, SEM, mechanical testing, four-point probe resistivity, and humidity response establishes a quantitative structure–property–function experimental investigation. This comprehensive correlation demonstrates that 3 wt.% GO loading near the percolation regime enables simultaneous mechanical reinforcement, controlled resistivity modulation, and stable humidity response.

Compared with the previously reported 1 wt.% GO system [27], the present 3 wt.% GO membranes exhibit a higher operating resistance window, greater structural stiffness, and stronger electrical tunability while maintaining the same proton-assisted humidity-sensing mechanism. These features position 3 wt.% GO/CMC composites as advanced multifunctional materials for next-generation humidity sensors, flexible electronics, and environmentally responsive devices.

## 4. Conclusions

In this study, 3 wt.% graphene oxide/carboxymethyl cellulose (GO/CMC) composite membranes were fabricated and systematically investigated in terms of their structural, mechanical, electrical, and humidity-dependent properties. The results demonstrated that increasing the CMC content significantly influenced the organization and functional behavior of the composite membranes.

Fourier transform infrared spectroscopy indicated interactions between GO and CMC functional groups through hydrogen bond formation. X-ray diffraction analysis revealed a gradual shift in the main GO-related diffraction peak from 2θ = 12.41° to 10.21°, corresponding to an increase in the calculated inter-layer spacing from 0.71 to 0.87 nm. These diffraction changes suggest modification of the layered organization of GO within the composite membranes. Scanning electron microscopy showed the progressive coverage and dispersion of GO sheets within the polymer matrix together with increasing structural uniformity at higher CMC contents.

Mechanical testing demonstrated an increase in tensile strength from 6.6 to 17.8 MPa with the increasing CMC concentration. Electrical measurements showed that the dry-state resistivity increased from 5.8 × 10^6^ to 2.32 × 10^7^ Ω·m as the CMC content increased. The observed mechanical and electrical changes were consistent with the structural and morphological modifications identified by FTIR, XRD, and SEM analyses.

Humidity-response measurements demonstrated reversible resistance changes within the 20–90% relative humidity range, indicating sensitivity of the membranes to water adsorption and humidity-dependent electrical behavior.

Overall, the combined FTIR, XRD, SEM, mechanical, and electrical results indicate that the CMC content plays an important role in controlling the structure–property relationships of GO/CMC composite membranes. The developed membranes may be considered promising candidates for humidity-sensing applications and other functional polymer-based composite systems.

## Figures and Tables

**Table 1:** XRD peak position, inter-layer spacing, FWHM, and crystallite size of GO and GO/CMC composite membranes.

Sample	2θ (°)	D-Spacing (nm)	FWHM β (°)	Crystallite Size D (nm)
GO	12.41	0.71	1.497	5.6
Initial CMC	20.46	0.43 *	3.396	2.5
GO/CMC (0.03 g)	11.48	0.77	1.273	6.6
GO/CMC (0.06 g)	10.77	0.82	1.924	4.3
GO/CMC (0.15 g)	10.21	0.87	1.932	4.3

* The CMC peak is a polymer-related diffraction feature and is not assigned to GO inter-layer spacing.

**Table 2:** Mechanical properties of 3 wt.% GO and GO/CMC composite membranes.

Sample	CMC Content (g)	Maximum Tensile Strength (MPa)	Young’s Modulus (MPa)	Strain at Break (%)	Toughness (MJ/m^3^)
3 wt.% GO	0	6.6	314	2.89	0.111
GO/CMC	0.03	7.6	126	3.79	0.252
GO/CMC	0.06	12.8	568	2.12	0.295
GO/CMC	0.15	17.8	838	2.18	0.512

**Table 3:** Comparison of mechanical properties for 1 wt.% and 3 wt.% GO systems.

System	Maximum Strength (MPa)	Young’s Modulus (MPa)
1 wt.% GO	2.3	23
1 wt.% GO/CMC (0.15 g)	14.3	143
3 wt.% GO	6.6	314
3 wt.% GO/CMC (0.15 g)	17.8	838

**Table 4:** Dry-state resistivities of GO and GO/CMC composite membranes (20% RH).

Sample	CMC Content (g)	Resistivity (Ω·m), 1 wt.% GO	Resistivity (Ω·m), 3 wt.% GO [27]
GO	0	1.51 × 10^6^	5.80 × 10^6^
GO/CMC	0.03	6.46 × 10^6^	8.70 × 10^6^
GO/CMC	0.06	9.27 × 10^6^	1.45 × 10^7^
GO/CMC	0.15	1.26 × 10^7^	2.32 × 10^7^

## Data Availability

The data presented in this study are available on request from the corresponding author.
